# First Parental Concerns and Age at Diagnosis of Autism Spectrum Disorder: A Retrospective Review from Malaysia

**DOI:** 10.21315/mjms2020.27.5.8

**Published:** 2020-10-27

**Authors:** Subhashini Jayanath, Sally Ozonoff

**Affiliations:** 1 Department of Paediatrics, Faculty of Medicine, University of Malaya, Kuala Lumpur, Malaysia; 2 Department of Psychiatry and Behavioral Sciences, UC Davis MIND Institute, Sacramento, USA

**Keywords:** autism spectrum disorder, delayed diagnosis, language delay, parent concerns, screen time

## Abstract

**Background:**

Autism spectrum disorder (ASD) is a neurodevelopmental disorder. This is the first study to examine first parental concerns in ASD in Malaysia. We examined: i) age and type of first parental concerns (AOC); ii) association between AOC and severity; iii) time lag between AOC and diagnosis; and iv) factors associated with diagnostic delay.

**Methods:**

Medical records of 366 patients (aged 1–18 years) with ASD, at the Developmental Paediatrics Clinic of University of Malaya Medical Centre (UMMC), Kuala Lumpur, were reviewed for this 16-month retrospective cohort study. A validated coding system was used for initial parent concerns. Severity was classified via the Diagnostic and Statistical Manual of Mental Disorders-5th edition (DSM-5) criteria. Time lag between AOC and age at diagnosis (AOD) was calculated. Potential predictors of delayed diagnosis were extracted.

**Results:**

Three-quarters (75.1%) of parents had concerns by 36 months. Speech/language/communication concerns were most frequent (60.1%). Number of first concerns was significantly correlated with severity (social communication/interaction, SCI [*P* = 0.019] and restricted, repetitive patterns of behaviours and/or interests/activities, RRB [*P* < 0.001]). AOC and AOD were significantly negatively correlated with SCI and RRB (*P* < 0.001). Medians; AOC: 24 months, AOD: 46 months and time lag: 17 months. Higher initial screen time was associated with diagnostic delay (*P* = 0.031).

**Conclusion:**

First parental concerns and AOD were comparable to studies across countries. Speech/communication delays may represent universal first parental recognition of ASD.

## Introduction

The prevalence of autism spectrum disorder (ASD) is steadily rising. According to the Centers for Disease Control and Prevention (CDC), the prevalence is 1 in 54 (1) for children aged 8 years. Prevalence rates vary considerably across regions (2). This variation likely has many causes, including medical resources and community services for evaluation (2). Another potential reason for regional variation is cultural differences in early parent concerns and how these are reported to healthcare professionals. A multinational study (3) found that parents in the United States of America (USA) tended to report repetitive movements in their children with ASD. Parents in Poland reported restricted interests, while Greek parents were more likely to notice unusual routines and rituals. Another cross-cultural study (4) in India, Japan and the United Kingdom reported cultural differences in the discriminatory value of parental responses to items on the Autism Spectrum Quotient (AQ)-Child.

Cultural differences in parental concerns in ASD are an important area of research. However, there is very little data from Southeast Asia. This study is the first of its kind in Malaysia. To date, there is no comprehensive Malaysian study on first parental concern(s), child’s age at first parental concern and time lapse to a definitive ASD diagnosis. This study explored the above factors in Malaysian paediatric patients with ASD and factors associated with delayed diagnosis.

First parental concerns are varied. Concerns regarding poor eye contact, pointing/gesturing, response to name and verbal communication are associated with an earlier diagnosis (5). Problematic child behaviour is associated with higher levels of parenting stress (6). Concerns that result in greater anxiety are likely to lead to earlier attempts at help seeking. It can be argued that an earlier presentation leads to more timely diagnosis.

The nature of first parental concerns may influence the clinician’s view of the urgency of a referral. Studying first parental concerns and their association with age at diagnosis (AOD) in a Malaysian cohort can inform the literature regarding culture-specific versus more universal factors. Elucidation of these factors can inform clinical practice and help with risk stratification with the aim of contributing to cross-cultural ASD research.

### Aims

This study aims to:

- Determine the age of first parental concern and the type of parental concern for paediatric patients who subsequently received a diagnosis of ASD
- Determine the association between first parental concern(s) and ASD severity
- Ascertain the time lag between the age of first parental concern and a diagnosis
- Identify factors associated with a delayed diagnosis (age > 36 months)

## Methods

### Study Design and Patient Recruitment

This was a retrospective cohort study of children with ASD. Total clinic visits at the Developmental Paediatrics Clinic of University of Malaya Medical Centre (UMMC), in Kuala Lumpur, during a 16-month time frame (January 2018–April 2019) were obtained. Patients diagnosed with ASD are routinely followed up at 6 months after diagnosis at the UMMC Developmental Paediatrics Clinic. All the patients who presented to the clinic during the 16-month period were included in this study. This included both those who presented either as new cases (diagnosed during the 16-month period) or follow-up cases (diagnosed prior to the 16-month period, but presented for their routine follow-up visits during the 16-month period of the study). If patients presented more than once during the study duration, only the first presentation during the study period was included to ensure that there were no duplicate cases.

The electronic medical records of all patients (aged 1–18 years at the time of diagnosis) with confirmed ASD, including those with comorbidities (e.g. epilepsy, sensory impairments), were reviewed. Patients younger than one year or older than 18 years were excluded. All patients had documented diagnostic confirmation of ASD by a developmental paediatrician using the Diagnostic and Statistical Manual of Mental Disorders-5th edition (DSM-5) (7) criteria. All consecutive cases meeting inclusion criteria were included. Universal sampling/census method was utilised for this study. Therefore, no sample size was calculated, since all the patients who fulfilled the inclusion criteria were included in the analysis. All the electronic notes were usable as a set clerking template has been in use in the Developmental Paediatrics Clinic since 2016. Therefore, incomplete documentation in the notes was not an issue.

Data collection was conducted via review of the electronic medical records (EMRs) of the doctors at the clinic. No parents or patients were interviewed. Information was extracted by a University of Malaya medical graduate, familiar with UMMC’s charting procedures and EMR system. She was trained in abstracting medical information, including parent concerns, from records and was continually supervised by the first author (SJ). Review of the quality and consistency of all data collected was done daily by SJ.

The Parent Concerns Questionnaire (8) was used to retrospectively code parent concerns documented in the EMR at the initial evaluation. SJ was trained in the coding system by the developers of the questionnaire and, after achieving over 90% reliability against a standardised training set, performed all coding. To ensure the reliability of the coding process, approximately 20% of concerns were double coded by the developers of the coding system (8) (97.5% reliability between coders).

Patient factors and socio-demographic data were obtained from the EMRs. A diagnosis after the age of 36 months was defined as a delayed diagnosis. The timing of a diagnosis was considered as the time between the parental first concern and that at which a definitive diagnosis of ASD was made. Those who were diagnosed prior to being seen at our Developmental Paediatrics Clinic were included in the study only if their diagnosis was verified at our centre, but the time of diagnosis taken was the earliest time at which a correct diagnosis of ASD was made. This was to ensure that the long waitlist at our clinic did not contribute to the time lag. Information on factors possibly associated with a delayed diagnosis were extracted from the EMRs, including ASD severity levels (social communication/interaction [SCI] deficits and restricted, repetitive patterns of behaviour (RRB) and/or interests/activities), initial screen time (hours per day) when the child was first introduced to screens, number of siblings with ASD, siblings with language/speech delay (yes/no) and age when medical attention was first sought (age at which the parents first sought an assessment by any doctor for the first parental concerns).

ASD severity was classified using the DSM-5 (7), on a scale which ranged from 1 (requiring support) to 3 (requiring very substantial support) for both SCI and RRB.

### Assessment Tool

The Parent Concerns Questionnaire (8) was used to code all parent concerns into 1 of 10 categories:

- No concerns
- Speech/language/communication concerns
- Social behaviour concerns
- Stereotyped behaviour concerns
- Motor concerns
- Medical concerns
- Behaviour/temperament concerns
- Unspecified concerns related to autism
- General developmental concerns
- Other concerns

This coding system has been used to quantify parental concerns for infants at high and low risk for ASD (8). In the current study, up to five parental concerns were coded. During the first clinic assessment, parents are routinely asked, “When were you first concerned about your child and what was your main concern then?” This was retrospectively extracted from the EMRs and recorded as the primary first concern.

### Statistical Analysis

Statistical analysis was performed using the IBM Statistical Package for Social Sciences (SPSS), version 25 (9). In order to determine whether there was an association between first parental concerns and ASD severity, Pearson correlation analysis was done. The assumptions for the correlation analysis were as follows: each variable was treated as a continuous variable and thus complied with the level of measurement assumption. There were related pairs, as each analysed factor was paired: SCI and RRB levels, total number of first concerns and SCI, total number of first concerns and RRB, most frequent primary concerns and SCI, most frequent primary concerns and RRB, age of first concern(s) (AOC) and SCI, AOC and RRB, AOD and SCI, AOD and RRB. There were no outliers beyond a value of ±3.29 standard deviation (SD) for any of the variables. This was tested by checking and confirming that the minimum and maximum values lay within this range. Linearity was established via a scatterplot.

Possible factors associated with a delay in diagnosis (increasing duration between the point of first parental concern and a diagnosis of ASD) were explored using regression analysis. Binary logistic regression was performed. The dependent variable was delayed diagnosis (age > 36 months). Therefore, the outcome was either delayed diagnosis (diagnosis at age > 36 months) or undelayed diagnosis (diagnosis at age ≤ 36 months). The observations were independent of each other because all the variables were not interrelated. There were no repeated measurements in our study; thus, this assumption was met. The other assumption was that a large sample size was required. Since there were 366 participants and the census method was employed, this assumption was also met. Factors analysed were ASD severity levels (SCI and RRB), initial screen time (hours per day) when the child was first introduced to screens, screen time (hours per day) at the time of a diagnosis of ASD, number of siblings with ASD, siblings with language/speech delay (yes/no), speech/language/communication concerns and age when medical attention was first sought (Table 4).

All continuous and categorical variables were included in the same logistic regression model. Factors were entered into a binary logistic regression model. The dependent variable (delayed diagnosis) was coded dichotomously. First, univariate logistic regression was done separately for each variable. All potential predictors (variables with *P* < 0.25) were then entered into a multivariate logistic regression model to confirm significance. Subsequently, via backward elimination, the least significant variables were removed one at a time until all predictors were *P* < 0.05. Remaining predictors were re-entered into the model to confirm significance. The final model with the remaining statistically significant predictor(s) (*P* < 0.05) was then tested. A *P*-value of < 0.05 was taken as significant. The percentage accuracy calculation (PAC) for the regression analysis was 77%.

## Results

### Study Population

Of the 366 participants included in the study, there were 300 (82.0%) boys and 66 (18.0%) girls.

### Age of First Concern

The majority (*n* = 275, 75.1%) had a concern by 36 months, comparable to that reported in other studies (1, 5, 10). Eleven parents (3.0%) could not recall the age of their first concerns. Thirty parents (8.2%) reported no concerns, having presented for an assessment due to concerns of others (extended family, doctors, teachers and neighbours).

The mean age of first concern was 29.3 months (SD = 16.5), similarly to previous reports in the USA (5) and China (11). The youngest AOC was 3 months. This child had a congenital cerebral malformation and initially presented with gross motor delay, which was the first parental concern. The oldest age of first concern was 120 months. The mode and median AOC were both 24 months. Table 1 shows the distribution of AOC and AOD.

### Type of Parental Concerns

The most frequent primary concern was speech/language/communication concerns (60.1%), which is consistent with other studies (8, 10) (Table 2). This was followed by social behaviour concerns (12.6%). Thirty (8.2%) parents did not have any concerns, double the percentage of a USA population study (5).

Table 2 shows the distribution of up to four additional concerns, without order of priority. There were 217 (59.3%) parents who had at least two first concerns and 112 (30.6%) had at least three first concerns.

### ASD Severity

The distribution of patients by severity levels is shown in Table 3. There was a significant strong positive correlation between SCI and RRB levels (Pearson correlation coefficient, *r* = 0.74, *P* < 0.001). Significant correlations were found between total number of first concerns and both SCI (*r* = 0.12, *P* = 0.019) and RRB (*r* = 0.23, *P* < 0.001) severity. Individual correlations between the most frequent primary first concerns and SCI and RRB severity levels were examined. There were no significant correlations between speech/language/communication concerns and either SCI (*r* = 0.02, *P* = 0.368) or RRB (*r* = 0.01, *P* = 0.424) severity. Similarly, for social behaviour concerns, there were no significant correlations with either SCI level (*r* = 0.004, *P* = 0.468) or RRB level (*r* = 0.07, *P* = 0.090). There was a significant negative correlation between AOC and both SCI (*r* = −0.28, *P* < 0.001) and RRB (*r* = −0.20, *P* < 0.001) severity. AOD was significantly negatively correlated with SCI (*r* = −0.33, *P* < 0.001) and RRB (*r* = 0.27, *P* < 0.001) severity.

### Time Lag between First Parental Concern and Diagnosis

The median age of first parental concern was 24 months, while the mean was 29.3 months. The median AOD was 46 months. This is slightly lower than what was found in the CDC study (1) (52 months). The mean AOD was 50.6 months (SD = 23.9), which was younger than studies in Nepal (12) (58 months), USA (5) (56.4 months) and Japan (13) (86.4 months), but older than the studies in China (11) (48 months). The youngest AOD was 12 months.

Mean time lag between first concern and diagnosis was 21.5 months, whereas median time lag was 17 months. The maximum time lag to a diagnosis was 101 months. This is consistent with the CDC (1) study, which reported that most parents had a first concern by 36 months but the median age for a diagnosis was 52 months, indicative of a significant time lag.

### Factors Associated with a Delayed Diagnosis (Age > 36 Months)

One hundred and fourteen (31.1%) participants were diagnosed by the age of 36 months, which is consistent with other studies (12, 13). Patient and socio-demographic factors were analysed to explore associations with delayed diagnosis (age > 36 months), as shown in Table 4. The cut-off age of 36 months was chosen because most parents in other studies had a first concern by this age (1).

## Screen Time

Lower initial screen time was associated with an 18% lower risk of a diagnosis after the age of 36 months (OR: 0.82; 95% CI: 0.68, 0.98; *P* = 0.031); that is, children with less screen time initially had a lower risk of delayed diagnosis. Since the current study was not longitudinal, the directionality of the relationship could not be determined. There was no significant association between the amount of screen time at the time of diagnosis and a delayed diagnosis.

## Age When Medical Attention was First Sought

Patients who were older when medical attention was first sought (the first time they presented to any doctor with developmental concerns) were more likely to have a delayed diagnosis (OR: 5.33; 95% CI: 3.35, 8.48; *P* < 0.001).

## Sibling(s) with ASD or Language Delays

Having a sibling with ASD was not associated with a delay in diagnosis. Having a sibling with a language delay was also not significantly associated with a delay in diagnosis. Another study (5) found that there was no statistically significant relationship between having a sibling with ASD and the likelihood of an early diagnosis. A follow-up study with a larger sample size could be done to further explore this relationship.

## Severity Levels

Neither SCI nor RRB levels were associated with a delayed diagnosis.

## Speech/Language/Communication Concerns

Speech/language/communication concerns were not associated with timing of diagnosis, which differs from a previous study in which verbal and nonverbal communication concerns were associated with an earlier ASD diagnosis (14).

## Discussion

This study highlights the types of first parental concerns, the age at which they arose, and factors associated with a delayed diagnosis in a large clinic-based cohort of children with ASD from Malaysia. Type of first parental concerns and AOD in this Malaysian sample were comparable to previous studies in different cultures (1, 5, 8, 10–12). Speech/communication delays were the most frequent first concern, as in previous studies conducted elsewhere (8, 10), suggesting that this may represent a universal, cross-cultural first parental recognition of ASD. There was a male predominance in this study, which is also consistent with other studies (1, 12, 15–17)

This study was confined to one centre and the study population mainly comprised those from urban areas, so there are limitations regarding extrapolation of the findings to the rest of Malaysia. However, it may be applicable to other paediatric outpatient populations with ASD in Malaysia from urban regions. It highlights the need for similar studies in the Southeast Asian region.

Higher ASD severity was associated with a younger AOC and an earlier AOD. Nevertheless, there was a time lag of almost 2 years (21.5 months) between first parental concern and a definitive diagnosis of ASD (i.e. the earliest age at which a correct diagnosis of ASD was made, even if this was prior to being seen by a developmental paediatrician at our centre). Reluctance of healthcare professionals to apply the diagnostic label of ASD may account for the almost two-year time lag between first parent concerns and diagnosis. It is plausible that parents may not have sought medical attention as soon as they could due to similar reservations. Since this is a retrospective study, these factors cannot be fully explored. Further studies should examine the reasons behind time lag, including delay in seeking medical attention, length of waitlists, or provider behaviours/reluctance to diagnose.

It was anticipated that having a sibling with ASD or one with language delay would have been associated with an earlier diagnosis, but contrary to expectations, these factors were not associated with timing of diagnosis. It was also surprising that speech/language/communication concerns were not associated with timing of diagnosis. It would have been expected that children whose parents had these concerns would present for an early assessment. It can be difficult, however, to distinguish isolated language delay from language delay in the context of ASD in very young children. If a language delay masked or overshadowed the social delays, the early language concerns might not have facilitated an earlier diagnosis.

There is a known association between excessive use of screen devices by toddlers and impaired language, communication and play/interaction skills (18, 19). However, the direction of the relationship and causality is uncertain. The American Academy of Paediatrics guidelines stipulate that screen time for children less than 18 months should only comprise of video chats, co-viewing must be practised for those 18–24 months and for ages 2–5 years screen time should not exceed one hour per day (20). In our study, the highest amount of screen time exceeded 10 hours per day and none of the children had zero screen time. We found that higher levels of initial screen time were associated with a delayed diagnosis. It is possible that symptoms of ASD, such as impaired language/communication skills, interpersonal skills and eye contact, are masked by excessive attention to screens. Thus, they present later and have a delayed diagnosis. Another explanation for the increased risk of a delayed diagnosis with increased initial screen time is children’s screen time practices are often a reflection of parental screen time practices (21). Increased use of screen devices by parents can impact parent-child interaction and quality time (22). This would affect the likelihood of parents’ noticing the non-specific early signs of ASD. It would be informative for future studies to assess whether increased parental media usage is associated with increased risk of a delayed diagnosis.

Communication and social interaction concerns are core features of ASD. The emergence of these first symptoms may be masked by excessive screen time. Limiting screen time could, potentially, reduce the time lag to a diagnosis. Screen time is a modifiable factor that can be ameliorated via effective public health awareness campaigns. Maternal and Child Health Clinic staff can play a role in advocating a restriction of young children’s screen time.

A limitation of this study is that all patients with confirmed ASD (including those with comorbidities like epilepsy and visual or hearing impairments) were included in the sample. This may have affected the median age of first concerns, since such patients may have presented earlier with parent concerns.

There are pertinent implications for practice:

- Since speech/language/communication concerns were the most frequent first concerns, clinicians should consider ASD, as well as language disorder, in any child presenting with speech-language delays
- Since number of first concerns was significantly associated with symptom severity, children with a higher number of first concerns will need prompt evaluation
- Over 75% of parents had concerns about their child’s development before age 3. Clinicians need to recognise that any developmental concern of parents warrants early referral. Parents also need to be encouraged to seek medical attention
- Higher severity levels were correlated with larger numbers of concerns and a younger age of first concern. However, the association of severity level with a delayed diagnosis did not achieve a level of statistical significance. Even so, clinicians need to be vigilant for early signs in children with milder features of ASD, as they are more likely to present with less parental concerns

The current practice in Malaysia includes screening 18-month-olds using the Modified Checklist for Autism in Toddlers (M-CHAT) during Well-Child Government Health Clinic reviews (23). Those who warrant further assessment are referred to government paediatric clinics. Subsequently, they are referred to developmental paediatrics clinics if available. While this helps detect young children with features suggestive of ASD, a clear pathway is needed for children who are not seen in government clinics and are instead followed up at private centres. Provision of training of private healthcare professionals on the scoring of ASD screeners, such as the M-CHAT, is one way of increasing the likelihood of detection of children with possible ASD. Furthermore, for children younger than 18 months, parental first concerns are sometimes not formally addressed during routine checkups. Another limiting factor is the two-tier referral process. If these at-risk children were referred directly for specialised ASD assessments (clinical diagnostic assessments such as usage of the DSM-5 by developmental paediatricians at government developmental paediatrics clinics) instead of awaiting review at a general Paediatric Clinic followed by a referral to a Developmental Paediatrics Clinic, the wait times to diagnosis would be considerably shortened.

## Conclusion

Parent education via public health awareness is crucial in ensuring recognition of the early signs of ASD. In the Malaysian context, systematic reporting of parental concerns has not been previously undertaken. Using a standardised coding system like the Parent Concerns Questionnaire (8) may improve the awareness of clinicians that first concerns can be predictive of specific diagnoses. Delayed speech is assumed to be a normal variant by many parents and clinicians, and so prompt action is not always taken. Since concerns about speech/communication, social behaviour and general development were the three most common parent worries in this study, these first concerns should prompt more in-depth and earlier specialist assessment. By improving the level of public awareness in Malaysia, Asia and across the world, young children with possible ASD or other developmental disorders will have a better chance of being seen by trained professionals earlier.

This study is the first to explore parental first concerns in Southeast Asia. The present results largely replicate previous studies done in other parts of the world (1, 5, 8, 10, 13). Speech/language/communication concerns were the most frequent first concerns, as was the case for two studies in the USA (8, 10). Thus, this category of concerns may represent a universal first parental concern for ASD, regardless of culture or geographical region. It would be important to replicate this study in other countries to determine whether this finding is consistent across diverse cultures. The utility of this would be in the development of questionnaires that target culturally neutral first parental concerns. The overarching goal would be to achieve a shift in the AOD to a progressively younger age, as this would enable access to early intervention programmes that optimise long-term outcomes in ASD.

## Figures and Tables

**Table 1:** Distribution of patients according to the age of a first concern and age of diagnosis, *n* = 366

Age (months)	Number, *n* (%)
**Age of AOC (months)**	
0–12	46 (12.6)
1–24	147 (40.2)
25–36	82 (22.4)
37–48	23 (6.3)
49–60	12 (3.3)
61–72	7 (1.9)
73–84	7 (1.9)
85–96	1 (0.3)
No concern (or concern by non-parent)	30 (8.2)
Unclear regarding exact age of parental first concern	11 (3.0)

Total	366 (100)
**Age of diagnosis (months)**	
0–12	2 (0.5)
13–24	19 (5.2)
25–36	93 (25.4)
37–48	92 (25.1)
49–60	72 (19.7)
61–72	36 (9.8)
73–84	23 (6.3)
85–96	6 (1.6)
97–108	8 (2.2)
109–120	7 (1.9)
121–132	4 (1.1)
133–144	3 (0.8)
145–156	0 (0)
157–168	1 (0.3)

Total	366 (100)

**Table 2:** Distribution of patients according to the primary first concern^a^ (*n* = 366) and remainder concerns^b^

Primary first concern		Number (*n*)		Percentage (%)
No concerns		30		8.2
Speech/language/communication concerns		220		60.1
Social behaviour concerns		46		12.6
Stereotyped behaviour concerns		3		0.8
Motor concerns		5		1.4
Medical concerns		4		1.1
Behaviour/temperament concerns		21		5.7
Unspecified concerns related to autism		4		1.1
General developmental concerns		24		6.6
Other concerns		9		2.5

Total		366		100

**Remainder first concern(s)**	**Concern 2 *n* (%)**	**Concern 3 *n* (%)**	**Concern 4** ***n* (%)**	**Concern 5 *n* (%)**
Speech/language/communication concerns	55 (15.0)	14 (3.8)	7 (1.9)	3 (0.8)
Social behaviour concerns	86 (23.5)	47 (12.8)	29 (7.9)	13 (3.6)
Stereotyped behaviour concerns	19 (5.2)	16 (4.4)	10 (2.7)	9 (2.5)
Motor concerns	5 (1.4)	2 (0.5)	0 (0)	0 (0)
Medical concerns	4 (1.1)	1 (0.3)	2 (0.5)	2 (0.5)
Behaviour/temperament concerns	22 (6.0)	21 (5.7)	16 (4.4)	3 (0.8)
Unspecified concerns related to autism	11 (3.0)	4 (1.1)	1 (0.3)	0 (0)
General developmental concerns	7 (1.9)	3 (0.8)	1 (0.3)	1 (0.3)
Other concerns	8 (2.2)	4 (1.1)	1 (0.3)	3 (0.8)

Total	217 (59.3)	112 (30.6)	67 (18.3)	34 (9.3)

Notes:

a primary first concern: the concern ranked as the most important by parents;

b remainder concerns: up to four parental first concerns, other than the primary first concern

**Table 3:** Distribution of type of primary first concern according to SCI level and RRB level, *n* = 366

Primary first concern	SCI level			Total
	1	2	3	
No concerns (code: 1)	13	12	5	30
Speech/language/communication concerns (code: 2)	66	97	57	220
Social behaviour concerns (code: 3)	15	18	13	46
Stereotyped behaviour concerns (code: 4)	2	1	0	3
Motor concerns (code: 5)	0	4	1	5
Medical concerns (code: 6)	1	1	2	4
Behaviour/temperament concerns (code: 7)	10	6	5	21
Unspecified concerns related to autism (code: 8)	1	2	1	4
General developmental concerns (code: 9)	7	5	12	24
Other concerns (code: 10)	1	7	1	9

Total	116	153	97	366

**Primary first concern**	**RRB level**			**Total**
	**1**	**2**	**3**	

No concerns (code: 1)	21	5	4	30
Speech/language/communication concerns (code: 2)	90	97	33	220
Social behaviour concerns (code: 3)	16	20	10	46
Stereotyped behaviour concerns (code: 4)	1	2	0	3
Motor concerns (code: 5)	1	3	1	5
Medical concerns (code: 6)	1	3	0	4
Behaviour/temperament concerns (code: 7)	11	6	4	21
Unspecified concerns related to autism (code: 8)	2	1	1	4
General developmental concerns (code: 9)	10	6	8	24
Other concerns (code: 10)	5	4	0	9

Total	158	147	61	366

**Table 4:** Multivariate analysis of factors associated with a delayed diagnosis (age > 36 months)

Factor	Odds ratio	*P*-value	95% CI
Lower SCI level	0.62	0.121	0.34, 1.14
Lower RRB level	0.67	0.225	0.35, 1.28
Lower amount of Initial Screen Time (hours per day)	0.82	0.031*	0.68, 0.98
Sibling(s) with language/speech delay	3.30	0.220	0.49, 22.14
Speech/language/communication concerns	1.85	0.071	0.95, 3.61
Older age when medical attention was first sought for concerns	5.33	< 0.001*	3.35, 8.48

**Factors excluded after univariate analysis**	**Odds ratio**	***P*-value**	**95% CI**

Higher amount of screen time at diagnosis (hours per day)	1.03	0.776§	0.83, 1.29
Number of siblings with ASD	1.22	0.724§	0.40, 3.73

Notes:

* significant results;

§ not included in the multivariate analysis, as *P* > 0.25 in the univariate analysis
